# Development of Coffee Biochar Filler for the Production of Electrical Conductive Reinforced Plastic

**DOI:** 10.3390/polym11121916

**Published:** 2019-11-21

**Authors:** Mauro Giorcelli, Mattia Bartoli

**Affiliations:** Politecnico di Torino, C.so Duca degli Abruzzi 24, 10129 Torino, Italy; mattia.bartoli@polito.it

**Keywords:** electrical properties, mechanical properties, recycling, epoxy resin

## Abstract

In this work we focused our attention on an innovative use of food residual biomasses. In particular, we produced biochar from coffee waste and used it as filler in epoxy resin composites with the aim to increase their electrical properties. Electrical conductivity was studied for the biochar and biochar-based composite in function of pressure applied. The results obtained were compared with carbon black and carbon black composites. We demonstrated that, even if the coffee biochar had less conductivity compared with carbon black in powder form, it created composites with better conductivity in comparison with carbon black composites. In addition, composite mechanical properties were tested and they generally improved with respect to neat epoxy resin.

## 1. Introduction

Anthropogenic waste stream management is one of the main unresolved problems of industrialized societies [1,2]. In the food waste sector, coffee residuals could be considered not only a waste material but a resource. Recently Christoph Sänger [3] reported that worldwide coffee production was 159.7 million of bags in crop year 2017/18 (about 9.6 MTons), with a mean of 5 kg/capita per year in traditional markets (Germany, Italy, France, USA and Japan) and an increasing consumption in emerging markets (South Korea, Russia, Turkey and China). The coffee waste stream becomes a relevant problem not only after consumption but also during the wet processing of coffee beans when 1 ton of fresh berries results in only about 400 kg of wet waste pulp. Several solutions have been proposed to solve the problem of waste coffee biochar, such as the production of biogas [4] and flavours [5], use as filler in ceramics [6] or as absorbent for the removal of basic dyes from aqueous solutions [7]. Coffee wastes have been also used as feedstock for pyrolytic conversion producing hydrogen-rich gas [8] and fuel-quality biochar [9]. Biochar has been used not only as solid fuel but also as high performance material [10,11], as a flame retardant additive [12,13], for electrochemical [14] and energy storage applications [15] and for production of composites [16,17,18,19].

Traditionally, in the realm of carbon fillers in polymer composites, carbon black (CB) plays the main role especially in the automotive field with an estimated consumption of 8.1 MTon/year according to data released by the International CB association [20]. CB has been used for producing conductive composites [21] but, as recently reported by Quosai et al. [22], coffee-based biochar also shows remarkably conductive properties. Furthermore, coffee biochar production has an indisputable advantage if compared with CB. Coffee biochar production uses a food waste stream while oil-based feedstock is required for CB production. This decreases the environmental impact of the production process [23,24,25].

Among different polymers, in this work we focused our attention on epoxy resins doped with these two carbon fillers. As is well known, epoxy resin is a thermoset polymer widely applied in the field of coatings [26], adhesives [27], casting [28], potting [29], composites [30], laminates [31] and encapsulation of semiconductor devices [32]. Epoxy resins are used intensively because of their peculiar properties such as high strength, good stiffness, good thermal stability and excellent heat, moisture and chemical resistance [33,34]. Another, unneglectable advantage of epoxy resin is the possibility of being dispersed into the cross-linked polymeric matrix additives, such as micro-encapsulated amines [35,36], that could be realised after material failure promoting the self-healing process of the epoxy composite [37].

In the field of composites materials, production of conductive reinforced plastic materials has attracted an increasing interest in the last few decades [38,39]. Large-scale application fields deserve particular attention. For example conductive epoxy resin has a large-scale application in the field of coatings and adhesives [40]. In these large-scale applications, filler cost is a crucial issue. Epoxy resins have been used as a polymeric host for plenty of carbonaceous materials for the production of conductive reinforced materials [41,42,43,44], but the cost of carbon filler has to be take in account. High-cost carbon fillers such as carbon nanotubes and graphene are problematic for large-scale applications. These carbon fillers induce an increment of its electrical and mechanical properties in the host polymer matrix [45,46,47,48] but are not a suitable choice for industrial scale production. This is mainly due to the high-cost, up to 300 k$/kg [49], and the problem of low productivity of the plants is well known [50]. Thus, low cost carbon fillers which are not derived from fossil fuels, such as CB, are a topic of relevant interest. 

In this study, we investigated the use of biochar derived from pyrolytic conversion of the coffee waste stream, such as low cost carbon fillers derived by recycling materials. Results were compared with CB-based composites. Mechanical properties were also investigated for full composite characterization.

## 2. Materials and Methods 

### 2.1. Carbonaceous Materials Preparation and Characterization

Exhausted coffee powder was selected as a real case study. It was collected from Bar Katia (Turin, Italy) supplied by Vergnano (Arabica mixture). Coffee was collected and dried at 105 °C for 72 h. Coffee samples (100 g) were pyrolyzed using a vertical furnace and a quartz reactor, heating rate of 15 °C/min and kept at the final temperature (400, 600, 800 and 1000 °C) for 30 min in an argon atmosphere. Samples were named as C400, C600, C800 and C1000 respectively. Biochar was grinded using a mechanical mixer (Savatec BB90E) for 10 min in order to decrease the particle size. Commercial CB (VULCAN^®^ 9 N115) was used to compare with coffee biochar. 

Ash contents of coffee and carbon-based materials (biochars and CB) were evaluated using a static furnace set at 550 or 800 °C respectively for 6 h. 

All samples were investigated from morphological point of view using a field emission scanning electrical microscopy (FE-SEM, Zeis SupraTM 40, Oberkochen, Germany). The microscope was equipped with an energy dispersive X-ray detector (EDX, Oxford Inca Energy 450, Oberkochen, Germany) that was used to explore the carbon composition of biochars.

Particle size distribution of carbon fillers was evaluated using a laser granulometry (Fritsch Analysette 22, Idar-Oberstein, Germany) after a dispersion in ethanol and sonication in an ultrasonic bath for 10 min. 

Coffee, biochars and CB were analysed through FT-IR (Nicolet 5700, Thermoscientific, Waltham, US) on attenuated total reflectance (ATR) mode (Smartorbit, Thermoscientific) in the range from 500 to 4000 cm^−1^.

Biochars and CB were analysed through Raman spectroscopy using Renishaw^®^ Ramanscope InVia (H43662 model, Gloucestershire, UK). 

### 2.2. Composites Preparation

Biochar, derived from coffee, and commercial CB containing epoxy composites were produced using a two component bis-phenol A (BPA) diglycidyl resin (CORES epoxy resin, LPL). Carbonaceous filler (15 wt. %) were dispersed into epoxy monomer using a tip ultrasonicator apparatus (Sonics Vibra-cell) for 15 min. After the addition of the curing agent, the mixture was ultrasonicated for another 2 min and left into the moulds for 16 h at room temperature. A final thermal curing was performed using a ventilated oven (I.S.C.O. Srl “The scientific manufacturer”) at 70 °C for 6 h.

### 2.3. Electrical Characterization

**Figure 1:**
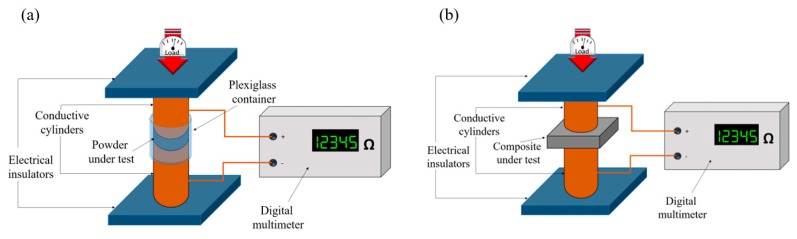
Sketch of measurement set-up for conductivity study of (**a**) carbon fillers and (**b**) composite.

The measurement set-up was derived from Gabhi et al. [51] and is sketched in Figure 1a for fillers and Figure 1b for composites. The instrument was composed of two solid copper cylinders, 30 mm in diameter and 5 cm in length, encapsulated in a hollow Plexiglas cylinder with a nominal inner diameter of 30 mm in the case of filler electrical characterization. In this configuration, the inner diameter was slightly higher so that it was possible to force the copper rods inside the Plexiglas cavity and the upper rod could slide inside the cylinder during the measurement. This arrangement created an internal chamber between the two cylinders, where the carbon powder could be inserted. In the case of composites, the Plexiglas cylinder was removed and the sample was positioned between the aligned copper cylinders. The electrical resistance of the powders or composites was measured at increasing loads (up to 1500 bar) applied by a hydraulic press (Specac Atlas Manual Hydraulic Press 15T). Electrically insulating sheets were placed between the conductive cylinders and the load surfaces in order to ensure that the electrical signal passed through the sample. The resistance of the carbon fillers was measured using an Agilent 34401A multimeter.

### 2.4. Composites Mechanical Characterization

Carbonaceous materials containing composites were produced as dog-bone shaped according to the ASTM 638 procedure. Samples were tested using a mechanical stress test (MTS) machine (MTS Q-test10) in tensile test mode until break point. Data were analysed using a self-developed software compiled using Matlab. 

### 2.5. Data Analysis

Statistical analysis used were based on t-tests with a significance level of 0.05 (*p* < 0.05) were carried out using Excel™ software (Microsoft Corp.) and the “data analysis” tool. 

## 3. Results

### 3.1. Carbonaceous Materials Characterization

Pyrolysis of spent grounds coffee proceeded according to the mechanism reported by Setter et al. [52]. The main mechanisms that occurred during the degradative processes were those related with decomposition of the small lignin fraction [53] and the most abundant polysaccharides (i.e., cellulose and hemicellulose) [54] with the formation of bio-oils rich in anhydrosugars, furans and acetic acid with trace of aromatics [55,56,57].

**Figure 2:**
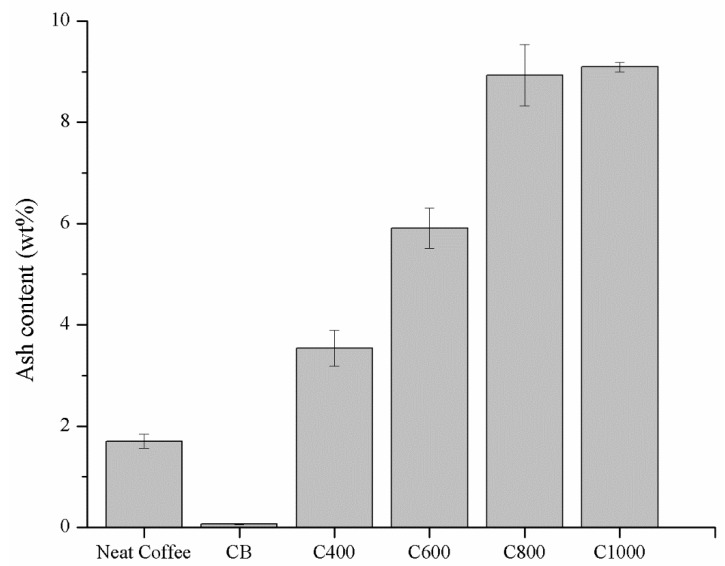
Ash contents of neat coffee, carbon black (CB) and coffee biochar samples heated at 400, 600, 800 and 1000 °C (C400, C600, C800 and C1000 respectively). Columns marked with different letters were significantly different (*p* < 0.05).

Ash content of feedstock and carbonaceous materials were preliminary investigated and summarized in Figure 2.

The ash content of neat coffee was 1.70 ± 0.14 wt. % and it increased with temperature increments reaching a value around 9 wt. % in the case of C800 (8.92 ± 0.61 wt. %) and C1000 (9.09 ± 0.09 wt. %). As expected, CB showed a very low ash content (0.07 ± 0.01 wt. %) according to Medalia et al. [58] mainly as oxides. Ash content increment at higher temperatures was imputable to advance pyrolytic degradation of the organic matrix leading to the concentration of inorganic residue [59] that did not undergo any temperature induced degradation. 

**Figure 3:**
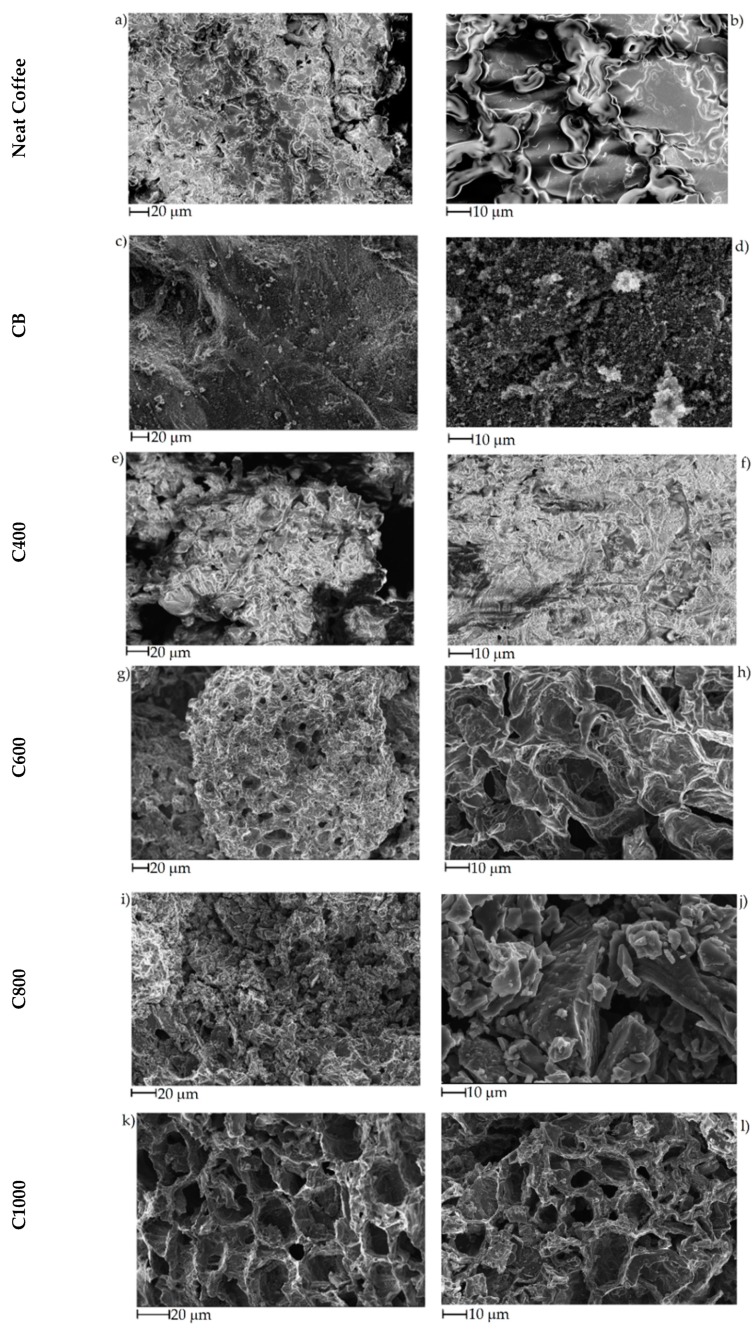
FE-SEM captures of (**a**,**b**) neat coffee, (**c**,**d**) CB, (**e**,**f**) C400, (**g**,**h**) C600, (**i**,**j**) C800 and (**k**,**l**) C1000.

The effect of pyrolytic temperature on biochar morphology was studied using FE-SEM as shown in Figure 3. Neat coffee displayed flaked collapsed structures (Figure 3a, b) that was retained by C400 after pyrolysis at 400 °C (Figure 3e,f). With the increase of temperature to 600 °C lead to the formation of porous structures with average diameters close to 30 μm separated by carbon lamellae with a thickness around 1 μm (Figure 3g,h). At 800 °C, the biochar recovered lost the structure due to the massive release of volatile organic matters during the overall pyrolytic process that induced the collapse of carbonaceous structures together with an improved grindability [60]. At 1000 °C, the increased temperature allowed the massive formation of carbon–carbon bonds that promoted the stabilization of the porous architecture with nanoscale lamellae structures. CB showed a typical highly aggregate spherule-based shape with average diameter of single particles around 50 nm.

**Figure 4:**
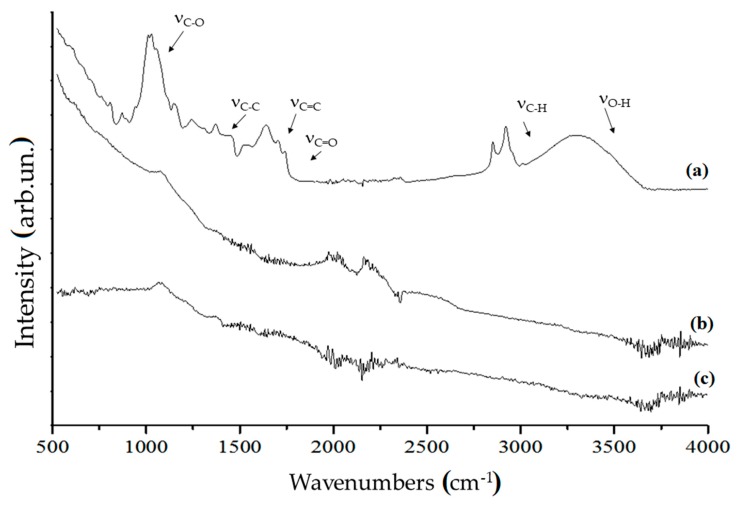
FT-IR spectra (ATR mode) of (**a**) neat coffee, (**b**) coffee biochar produced at 1000 °C and (**c**) CB in the range of 500–4000 cm^−1^.

Organic component of significant carbonaceous materials was also analysed using both FT-IR and Raman spectrometry techniques. Among carbonaceous materials, we reported neat coffee, C1000 and CB. Results are shown in Figure 4. 

The FT-IR spectrum of neat coffee showed the broad band of ν_O–H_ (3300–3500 cm^−1^), the bands of saturated ν_C–H_ (2850–2950 cm^−1^), ν_C=O_ (1710–1741 cm^−1^) due to the carboxylic functionalities, ν_C=C_ (1540–1638 cm^−1^) due to the presence of aromatic structures, saturated and unsaturated δ_C–H_ (1370–1440 cm^−1^), saturated ν_C–C_ (1243 cm^−1^), ν_C–O_ (1030–1148 cm^−1^) and out-of-plane δ_O–H_ below 700 cm^−1^. Those bands clearly identified a lignocellulosic derived matrix with massive presence of polysaccharides and aromatics. C1000 did not show any of the characteristic bands of organic matrix but show an envelope of bands below 1800 cm^−1^ due to carbon skeletal movements. Contrary, CB showed low bands intensity below 1000 cm^−1^ due the lower variety of carbon structure embedded into particles.

**Figure 5:**
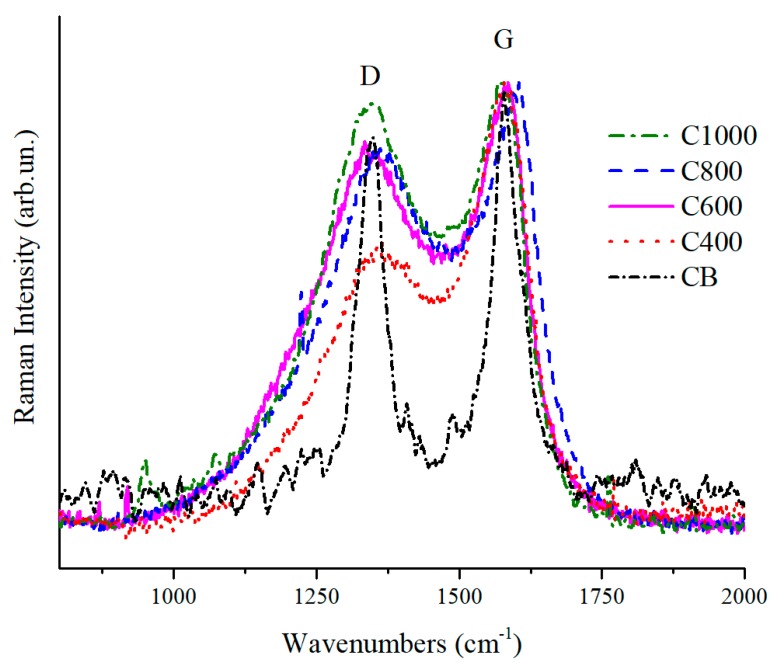
Magnification of Raman spectra in the range from 800 to 2000 cm^−1^ of C400, C600, C800, C1000 and CB.

Raman spectra normalized on G peak are shown in Figure 5. Coffee biochars had the typical profiles of amorphous materials [61] in contrast to CB which was more graphitic. The graphitic structure for CB could be observed by the deep gorge between D and G peaks and their shaped structure. An increase of *I*_D_/*I*_G_ ratio was evident for biochars moving from a pyrolytic temperature of 400 to 1000 °C. This increase of *I*_D_/*I*_G_ ratio could be ascribed to the progressively loss of residual functional groups with the increase of temperature. This observation was also supported by the decrease of fluorescence [62]. Due to the loss of less intense parts of these weak interactions, biochar underwent an appreciable disorganization together with aromatic structure formation, in particular up to 600 °C, without the completion of a proper graphitization process that occurs at higher temperature [63].

The evolution of biochar structures due to temperature increment could be monitored though Raman according to Ferrari et al. [61]. Accordingly, the D peaks (Figure 5) showed wave numbers close to 1350 cm^−1^ that is typical of transition from amorphous carbon to nanocrystalline graphite. At the same time the biochar G peaks showed wavenumbers close to 1580 cm^−1^ with exception of C1000. This last showed a G peak at 1600 cm^−1^ due the high amount of nanocrystalline domains not yet rearranged in the ordered structure [64]. 

**Figure 6:**
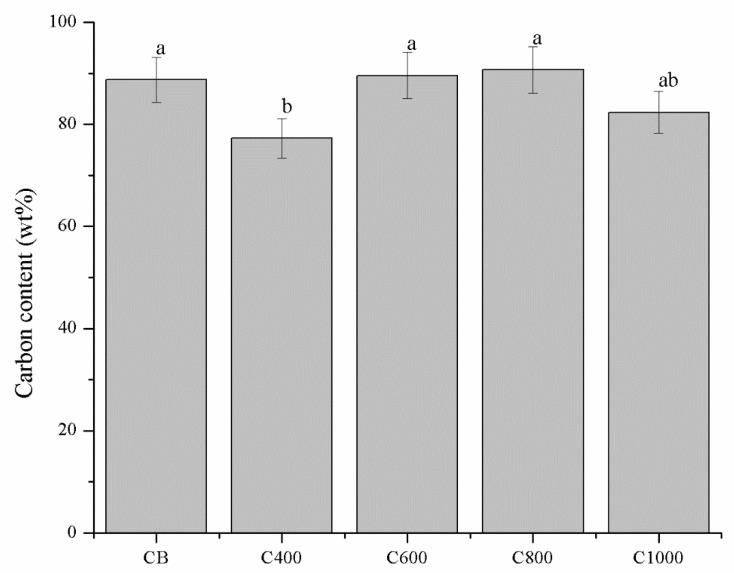
Carbon content of biochars and CB. Evaluated though energy dispersive X-ray (EDX) analysis. Error values were reported as 5% of the detected values according with Laskin et al. [65]. Columns marked with different letters are significantly different (*p* < 0.05).

The above mentioned consideration was also supported by EDX analysis that showed the carbon content that significantly increased from C400 to C600–C1000 while oxygen content decreased. Carbon content of C600–C1000 were not significantly different from CB even if CB showed a more ordered structure. This support the hypothesis that the driving force of the biochar enhanced conductivity is the reorganization of nanocrystalline domains and not merely the carbon content, shown in Figure 6. Traces of Mg, P, K and Ca were also detected.

### 3.2. Composites Characterization

#### 3.2.1. Electrical Characterization of Carbonaceous Filler and Composites 

**Figure 7:**
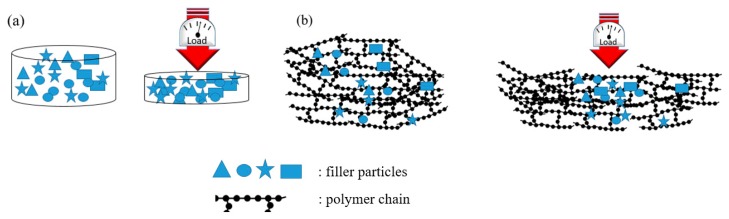
Behaviour of (**a**) powders and (**b**) composites during compression.

The set-up shown in Figure 1a was used for biochar powders electrical characterization. Around 3 g of carbonaceous powder, which creates a few millimetres distance between copper cylinders was positioned in the chamber. After the closure of the chamber a pressure was applied with the aim of compacting the powder. The pressure range was from 0 to 1500 bar (step of 250 bar). For each step the stabilized value of resistivity was registered such as the distance between the copper cylinders. The same procedure was repeated for composites of few millimetre thickness. Carbonaceous powders and composites decreased their resistance value during compression until they reached a plateau when high pressure was reached. The decreasing of resistance value could be correlated with the decreasing of space between carbon particles as sketched in Figure 7. In the case of powders, the void among particles collapsed with a production of compact carbon agglomerate as shown in Figure 7a. In the case of composites, Figure 7b shows the mechanism where the polymer chains flow let the carbon particles situate. 

The resistance value, R, with the value of surface S and distance l between copper surfaces were used in Ohm law (σ = l/*RS*) to evaluate the conductivity σ. The conductivity value of carbon powder and composites were evaluated following this procedure:

(1) A starting value of conductivity was evaluated without any sample in order to measure the value of resistance of the system. This value was subtracted to the resistance value read with samples.
(2) The same quantity of carbon powders (CB and biochar) were positioned between copper cylinders and kept by the Plexiglas hollow cylinder. The measurement was repeated several times in order to have a reliable value.
(3) Composites were positioned between copper cylinders, in this case the Plexiglas hollow cylinder was not necessary and the value of conductivity was measured in different sample portions.

**Figure 8:**
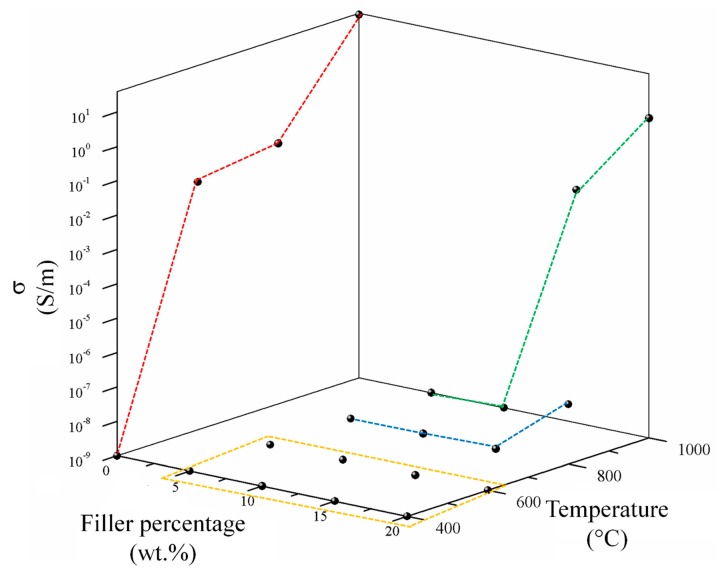
Trends of composites conductivity as function of filler percentage. Red line represents the biochar conductivity trends while yellow, blue and green show respectively the percolation curves of composites containing C400–C600, C800 and C1000.

Preliminary results are shown in Figure 8 showing the conductivity of the biochar powders (red line) and percolation curves of related composites.

C400 did not show an appreciable conductivity while an increment of pyrolytic temperature up to 600 °C induced a conductivity of up to 0.02 S/m. Further increments of processing temperature up to 800 and 1000 °C led to a conductivity of up to 0.04 and 35.96 S/m. This remarkable increment of conductivity between 800 and 1000 °C was due the enlargement of aromatic region formed as consequence of high temperature carbonization [64]. This deeply affected the electrical behaviour of related composites. Consequently, C400 and C600 containing composites were not conductive for all the range of filler percentage investigated. CB composites were not conductive until the filler concentration of 15 wt. % reaching a conductivity of 5.4 × 10^−8^ S/m with a filler loading of 20 wt. %. C1000 composites showed the best performances showing a detectable electrical conductivity with a 15 wt. % of filler and reaching a conductivity of 2.02 S/m with a filler loading of 20 wt. %.

**Figure 9:**
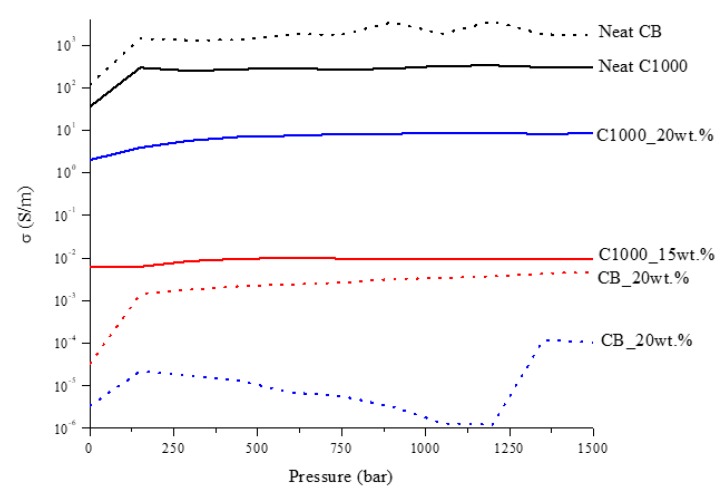
Trends of CB and C1000 powders and related composites conductivity as a function of pressure applied.

Accordingly, with these data, electrical properties of C1000 and C1000 containing composites were studied under a wide range of static pressures comparing with the related CB and CB composites as shown in Figure 9.

**Figure 10:**
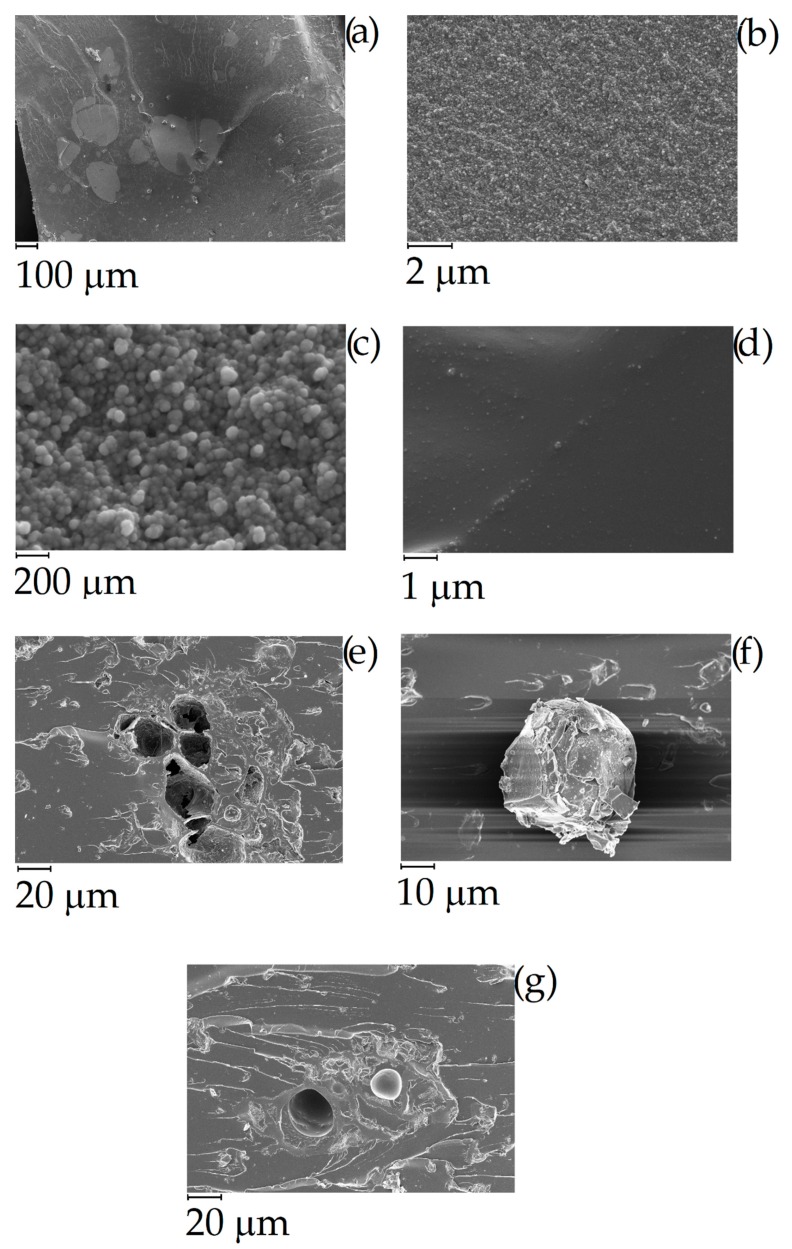
Field emission scanning electrical microscopy (FESEM) of cryo-fractured (**a**–**d**) CB and (**e**–**g**) C1000 containing composites with a filler loading of 15 wt. %.

**Figure 11:**
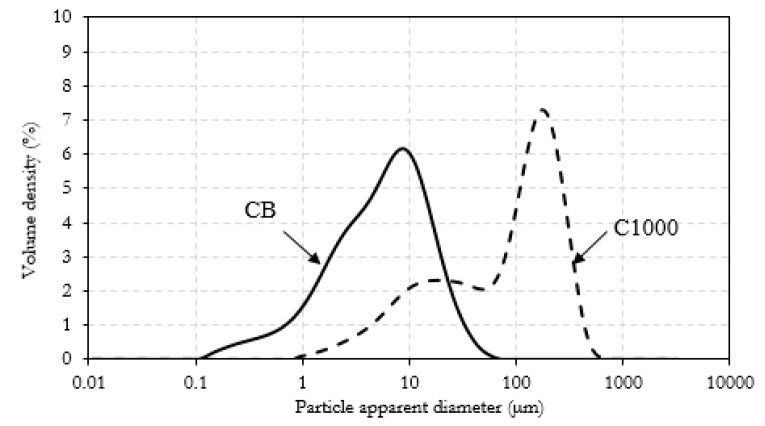
Particle size distribution for CB and C1000.

CB powder reached a conductivity around 1700 S/m while in the same conditions C1000 reached a conductivity of 300 S/m. Composites containing of CB and C1000 were conductive but the results showed a different trend compared with the relative powders. CB 15 wt. % reached the value of 4 × 10^−3^ S/m and its conductive value showed an influence of applied pressure in the first compression movement. C1000 15 wt. % reached to 10^−2^ S/m, with an increment around one order of magnitude if compared with CB 15 wt. %. This difference was more relevant for a filler concentration of 20 wt. %. In this case, the conductivity of CB-based composites dropped down to 10^−5^–10^−4^ S/m in contrast to C1000 which reached ~10 S/m. The high conductive value for coffee biochar could be due to more uniform filler dispersion inside epoxy resin. Dispersion of the filler inside the epoxy matrix was investigated through FE-SEM (Figure 10) after samples were cryo-fractured using liquid nitrogen and compared to composites with a filler loading of 15 wt. % due to the similarity of conductivity. CB containing composites showed a dark and clear area (Figure 10a) with different compositions. The clear ones were rich in CB aggregates (Figure 10b,c) while the darkest were poor (Figure 10d). C1000 containing composites showed smooth surfaces with holes (Figure 10e) due the expulsion of embedded C1000 particles during the fracturing (Figure 10e) as clearly shown in Figure 10g. Particles size analysis (Figure 11) showed clearly that C1000 was composed by two particle populations, one around 100 μm and one around 20 μm. Considering the average size of C1000 particles into the composites was reasonable it was assumed that the bigger ones underwent a disruption during the ultrasonication forming small sized well-dispersed particles. CB particles size showed also that it would be more appropriate speaking of CB aggregates instead of single particles [66]. Aggregates could be justified also from Figure 10c where the CB single particles were less than 100 nm but they created agglomerates that also the particle size analysis (Figure 11) was not able to detect. 

#### 3.2.2. Composites Mechanical Characterization

**Figure 12:**
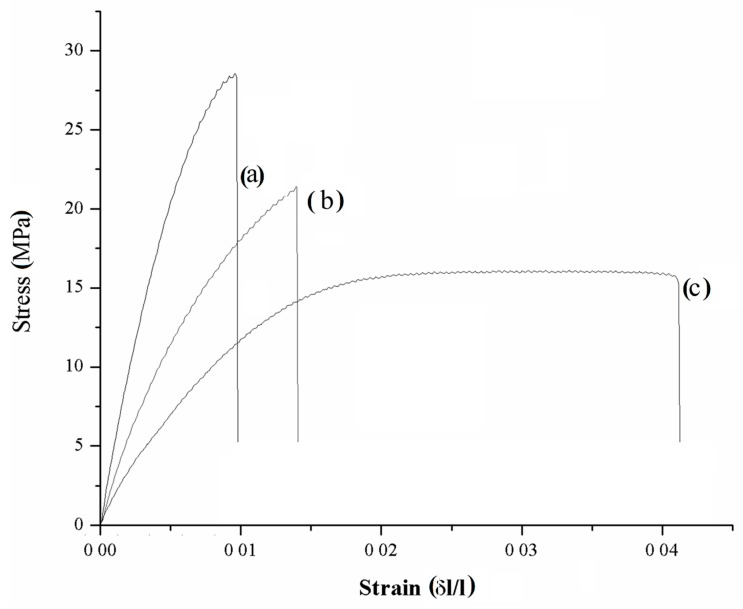
Stress–strain curves of composites containing 15 wt. % of (**a**) C1000, (**b**) CB and (**c**) neat resin.

With the aim of confirming mechanical consistence of samples, a stress–strain curve was investigated for composites of 15 wt. % for CB and C1000 compared with neat resin. Mechanical tests on dog-bones shaped samples are summarized in the Figure 12.

**Figure 13:**
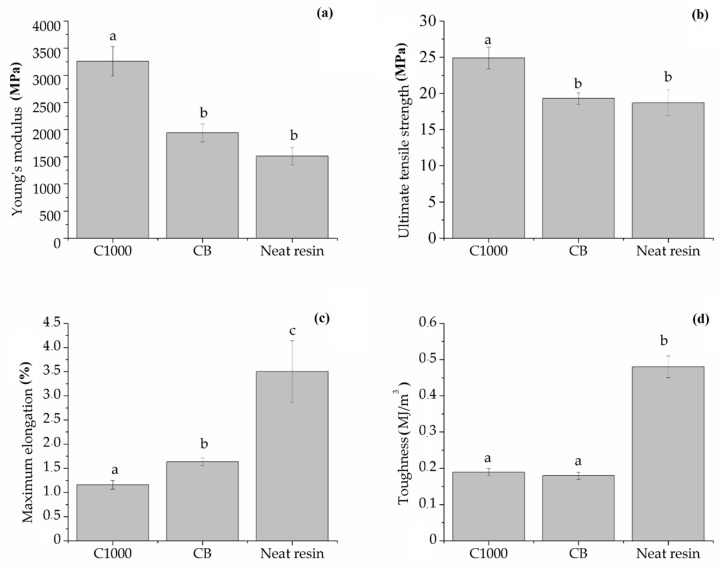
Summary of (**a**) ultimate tensile strength, (**b**) Young′s modulus, (**c**) toughness and (**d**) maximum elongation of neat resin, biochar and carbon-based composites. Columns marked with same letters were not significantly different (*p* < 0.05).

According to data report in Figure 13, maximum elongation of neat resin (3.50% ± 0.64%) was the highest compared with those of C1000 and CB containing composites (1.16% ± 0.09% and 1.63% ± 0.08% respectively). Neat resin showed also a remarkably higher toughness (0.48 ± 0.03 MJ/m^3^) compared with composites that showed values not significantly different from each other close to 0.18 MJ/m^3^. Young′s modulus (YM) showed a significant difference between C1000-based composites (3258 ± 273 MPa) and CB ones (1940 ± 163 MPa). These last values were quite close to those of neat resin (1510 ± 160 MPa) and a similar trend was observed with ultimate tensile strength with values of CB composites not significantly different from those of neat resin (both close to 19 MPa) and higher values for biochar-based composites (up to 24.9 ± 1.5 MPa).

Composites behaviour observed during the mechanical tests enlightened the different interaction between different carbonaceous filler with epoxy matrix with magnification of filler–resin interaction, and in the case of biochar-based composites with an increase brittleness and a reduced elongation.

As reported by Chodak et al. [39] about CB containing poly(propylene) composites, the formation of a diffuse particles network is detrimental for the mechanical properties. The same behaviour was observed in the production of CB-based composites which presented a decrement of Ultimate tensile strength compared with CB1000 ones. CB1000 were very close to the percolation threshold (Figure 7) and this induced a very relevant decrement of maximum elongation. Working below the percolation threshold allowed the preservation of some of the appealing properties of a brittle resin (i.e., high Young’s modulus and ultimate tensile strength) together with the magnification of electrical conductivity.

## 4. Conclusions

The coffee waste stream was efficiently used as feedstock for pyrolytic conversion at different temperatures. The effect of process temperature on the properties of biochar was investigated and it was observed that further increments of temperature improved the porous stability and conductivity of the material. This phenomenon was probably due to both the formation of new C–C bonds and to the rearrangement of graphitic and quasi-graphitic domains formed during pyrolysis as shown by Raman characterization. 

The most relevant result of this study was that even if neat biochar produced at 1000 °C showed less conductivity with respect to CB when it was dispersed in composite, the electrical properties of a composite containing coffee biochar were some orders of magnitude higher than composites containing CB. In the case of 20 wt. % of C1000, composites showed four orders of magnitudes more that composites containing 20 wt. % of CB. This could be ascribed to the uniform dispersion of coffee biochar, in contrast to CB which creates agglomerations. These agglomerations induced a non-uniform composite structure in the CB containing composites. Mechanical properties of composites with coffee biochar were verified and they were not compromised with respect to composites containing C1000, showing better UTS and YM. Both materials were more brittle than neat resin but C1000 showed some of the properties of high performances resins. Mechanical properties also showed a direct correlation with filler dispersion. Where the filler dispersion was uniform, the mechanical performances were improved. 

A new era could be at the door for carbon fillers in polymer composites. Considering the sustainability of coffee biochar production, the results reported show how biomass-derived carbon could be a sound replacement for oil-derived carbon fillers such as CB.
